# Assessment of Nitric Oxide Release In Vitro via Low-Level Daylight-Equivalent Blue or Red Light Irradiation

**DOI:** 10.3390/antiox15020232

**Published:** 2026-02-10

**Authors:** Gareth Hazell, Marina Khazova, Paul O’Mahoney

**Affiliations:** 1 UK Health Security Agency, Centre for Radiation, Chemical Climate and Environmental Hazards, Harwell Campus, Chilton, Didcot, Oxford OX11 0RQ, UK; 2 NIHR Health Protection Research Unit in Radiation Threats and Hazards, London SW7 2AZ, UK

**Keywords:** nitric oxide, nitrite, photobiomodulation, skin, cardiovascular disease

## Abstract

Conflicting evidence exists on whether blue or red light modulates nitric oxide (NO) release within skin cells. Using environmentally relevant doses, irradiances and spectral distribution akin to low-level sunlight, we provide evidence that broad-spectrum blue or red (including infrared) light fails to significantly increase NO release in skin cell monolayers compared to unexposed controls for each individual timepoint assessed. We discuss observed discrepancies between our work and recent photobiomodulation (PBM)-based studies presenting measurable upregulation, noting that significant NO induction typically requires high-powered light-emitting diodes (LEDs) or lasers used in clinical settings within a specific narrow spectral band. Thus, while our findings show no significant effect, they provide an important counterpoint for public health discussions on visible light exposure at terrestrial levels, particularly low-level exposures, between 15 and 30 min midday UK visible light exposure.

## 1. Introduction

Photobiomodulation (PBM) therapy was originally documented over 50 years ago using low-powered ‘ruby’ light to stimulate hair regrowth and wound healing in rodents [1]. Analogous to pharmacological intervention and guided by the Arndt–Schultz law, effects are governed by the precise control of light-based parameters such as energy, fluence, and irradiance for specific wavelengths via the use of laser and LED-based sources exerting effects through cellular chromophores [2]. A ‘therapeutic window’ is therefore derived under these conditions, stimulating cellular metabolism without causing inhibition or harm that (as the Arndt–Schultz law highlights) is induced at exposures outside the therapeutic window [2].

A key challenge in PBM research is the lack of consensus on the optimal parameters for achieving therapeutic effects, with reported fluences ranging from 0.5 to 50 J/cm^2^ depending on the tissue type, experimental setup, lamp type, and spot size incorporated into the study [2]. Visible light within low-dose environmental exposures may also elicit biological effects [2].

The generation of the redox-active mediator nitric oxide (NO) remains a focus within research in this area. Specifically, NO can harbour a range of positive responses within a therapeutic window, enhancing endogenous antioxidant defences and modulating inflammatory pathways [3]. Locally within the skin, positive effects contribute to enhanced wound healing, melanogenesis and lipogenesis, maintaining skin barrier integrity [4,5,6,7]. Remarkably, skin-driven generation also contributes to systemic cardiovascular benefits, facilitating extracellular cross-talk and modulating blood flow within arteries [8], implicated in hypertension [9,10]. NO induction in healthy individuals is mediated by endothelial secretion, which diffuses to adjacent vascular smooth muscle cells (VSMCs), eliciting a vasodilatory response [11]. Nitric oxide synthases (NOSs), enzymes responsible for NO production in the endothelium, play a critical role in this process [12,13]. However, NOS activity alters with age, with the levels of NO declining (due to uncoupling of the enzyme from dimeric form to monomeric), stimulating superoxide release and oxidative stress through enhanced peroxynitrite induction. This impairs local homeostasis and vascular function [12,13].

The liberation of NO by ultraviolet radiation (UVR) from salts held at high concentration within keratinocytes, the primary skin cells making up 90% of the epidermis, results in local and systemic effects via a non-enzymatic stimulus [14,15] and may be beneficial and have the highest impact for subsets of the population with decreased enzyme-based responses [12,13].

Recently, PBM studies have suggested that light-emitting diodes (LEDs) and lasers emitting longer wavelengths, specifically blue (400–500 nm), red visible light (620–780 nm) and near-infrared (780–1000 nm), may also facilitate NO release via non-enzymatic mediators [16,17]. Here, NO is derived from nitroso-based compounds within the epidermis and light causing NO release through mitochondrial function via the chromophore cytochrome c oxidase [16]. Thus, it is feasible that these secondary mechanisms, if potentiated through visible light in sunlight, may also supplement NO lost via NOS uncoupling as ageing progresses [16,17]. Should such a response be recapitulated via the exposure of the skin to visible light in sunlight (for example, in the morning or evening where significantly lower ultraviolet-B irradiance occurs outside the midday peak) [18,19], this represents an important consideration for public health, as UVR exerts a range of deleterious effects on skin cells under high or prolonged exposure, including the modulation of inflammatory pathways, the induction of photo-immunosuppression, enhanced oxidative stress and the acceleration of photoageing [20]. Moreover, UVR is recognised as a ‘complete carcinogen’ capable of both tumour initiation and promotion with numerous epidemiological studies demonstrating strong associations with an increased incidence of basal cell carcinoma, squamous cell carcinoma, and malignant melanoma [21].

In this study, we assess NO release in vitro by visible blue or red/infrared light derived from a filtered solar-simulated light source which mimics terrestrial sunlight levels. Here we opt for these exposures on the basis that previous work suggests that NO is induced with a negligible genotoxic effect [12,22]. We report no detectable increase in NO within the timepoints assessed when compared against a time-matched unexposed control.

## 2. Materials and Methods

### 2.1. Primary Cell Isolation and Culture

Primary keratinocyte, fibroblast and endothelial cell lines were isolated from two white-skinned (Fitzpatrick scale types I and II), non-related neonatal foreskins (≤1 month old) collected post-circumcision and grown in culture, as previously described [12,22]. Informed consent was obtained from all subjects in this study prior to tissue collection, under approval from the South-Central Berkshire B Ethics Committee (22/SC/0411, IRAS ID 31832) dated 15 December 2022. For experiments, cells were seeded at a density of 250,000 cells per well and cultured for four days to reach confluency.

### 2.2. Exposure of Cell Cultures to Light

**Figure 1:**
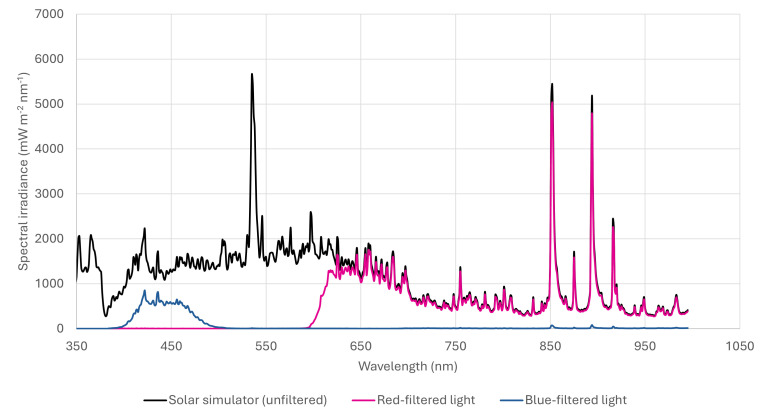
A schematic representation of the modified SOL-2 solar simulator’s (Dr Honle AG UV-Technologies) spectral output used to deliver daylight-equivalent visible light. Long-pass and band-pass optical filters were applied to isolate blue (400–510 nm), red (600–800 nm), and near-infrared (800–900 nm) spectral regions, indicated by the ‘blue-filtered light’ and ‘red-filtered light’ bands above. The filtered light setup incorporated continuous air flow to prevent thermal artefacts, while the placement of filters to allow for air flow minimised heat transfer and ensured homogenous irradiance across wells. Where dose differences exist between blue and red visible wavelengths, these reflect the relative levels naturally experienced during environmental visible light exposure.

At 95–100% confluency, visible light exposures were performed on cell monolayers following the removal of cell culture media and replacement with PBS +/+ (phosphate-buffered saline with calcium and magnesium) (CSR156 Appleton woods ™, Birmingham, UK). The removal of media was carried out at the stage of light exposure only as preliminary work, and other studies have shown that light can induce the photodegradation of media constituents such as tryptophan, resulting in the liberation of hydrogen peroxide (H_2_O_2_) and subsequent non-physiological oxidative stress in cell monolayers in turn also upregulating reactive nitrogen species (RNS) and NO [23,24]. Exposures were conducted using a SOL-2 solar simulator (Dr Hönle AG UV-Technologies ™, Zörbig, Germany), with emission in the blue and red spectra at levels similar to terrestrial sunlight. The device was modified to include an internal fan to dissipate heat. Desired spectral regions were isolated using a combination of long-wave and band-pass filters (Figure 1). Filters were mounted on shielded lids, providing a gap between the plate and filter for air flow to prevent excess heat, previously identified as arising from radiation absorbed by the filter when positioned too close to the cells [22]. Cell monolayers were exposed to a single exposure of either 4.86 J/cm^2^ blue light (400–510 nm) at an irradiance of 5.40 mW/cm^2^ or 20.32 J/cm^2^ (10.14 J/cm^2)^ red (600–780 nm) and 10.18 J/cm^2^ infrared (780–900 nm)) at an irradiance of 33.9 mW/cm^2^. This is approximately equivalent to between 15 and 30 min midday UK visible light. Following exposure to blue or red light, monolayers were compared against a corresponding unexposed control for nitric oxide induction. Negative controls were held at the same temperature as exposed samples but shielded from the effects of light.

Positive controls, known to induce NO release through increased florescence from probes DAF-FM and DAX-J2 [22], were incorporated into this study, via the use of the BIOSUN UV device (VILBER LOURMAT™, Paris, France). The positive controls were irradiated with 1 J/cm^2^ UV-B. The BIOSUN incorporates live dose and temperature monitoring, ensuring consistent dose delivery; thus the accurate delivery of positive controls was ensured.

### 2.3. Nitric Oxide Detection

The levels of NO were detected via the use of cell-permeable probes, DAF-FM diacetate (DAF-FM DA; D23844, Thermo Fisher Scientific, Waltham, MA, USA) and DAX-J2 Red (16301-AATB, AAT BIOQUEST™, Pleasanton, CA, USA), understood from the manufacturers’ protocols to be highly sensitive in terms of assessing NO production to a level of 3 nM. Probes were added via addition to cell culture media for 45 min to exposed, unexposed and positive controls. Media were then removed and replaced with PBS+/+ as at this point the probe was uptaken by cell lines. Cells were counterstained with either propidium iodide (P3566, Thermo Fisher Scientific, Waltham, MA, USA) or CELL-TOX green (G8741, PromoCell™, Heidelberg, Germany) added prior to flow cytometry but after visible light exposure to remove dead or dying cells. Exposed cell samples at each timepoint were accompanied by time-matched unexposed and positive controls. The use of alternate probes excited within the blue and red spectra after light exposure allowed the quenching of the probe (providing erroneous results) to be subverted when blue and red light were administered, as we experienced in previous work [22].

### 2.4. Statistical Analysis

Statistical analysis was conducted using GraphPad Prism^TM^, version 10.1.2 In this study, we were only concerned with changes in NO within individual timepoints, not between timepoints, cell types or red/blue light sources. Therefore cross-comparison was not made between these factors. Given that means were compared only within individual timepoints between the red/blue light source and their corresponding control, an ordinary one-way ANOVA with a mixed-effects model was used. Significance was set as *p* < 0.05. More details are available in the Supplementary Materials.

## 3. Results

**Figure 2:**
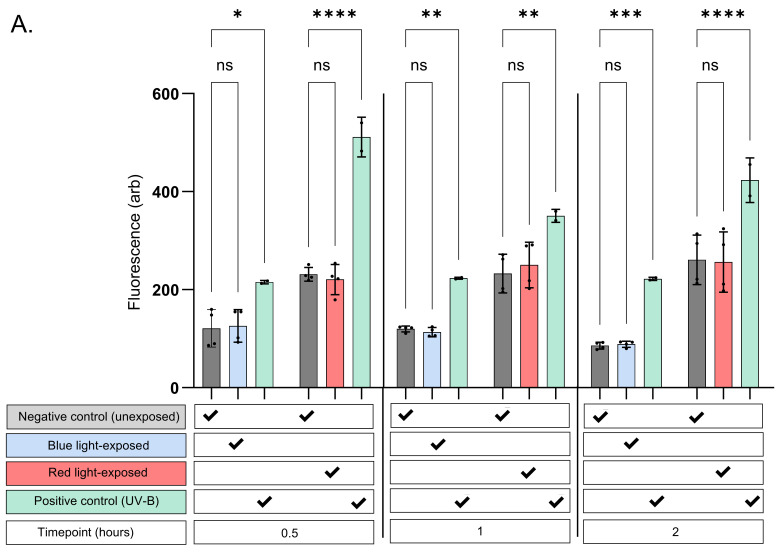

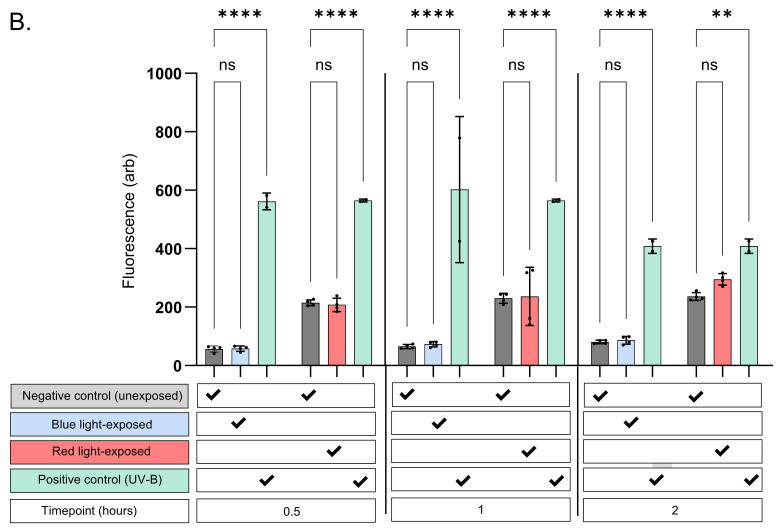

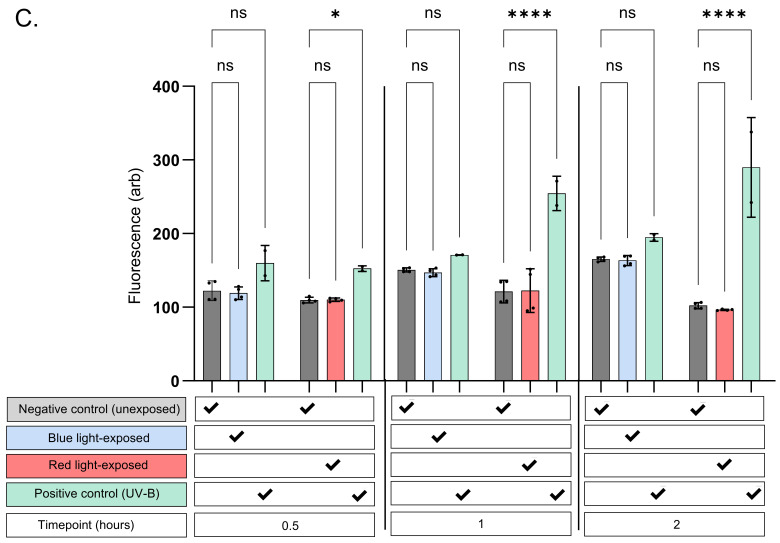
(**A**–**C**) Fluorescence recorded from flow cytometer (Y axis) from cell-permeable probes DAX-J2 Red and DAF-FM diacetate used to detect NO generation in cells exposed to blue (400–500 nm) and red/infrared (>600 nm) light, respectively (X axis). DAX-J2 fluorescence was recorded at peak emission of 609 nm, DAF-FM fluorescence at 515 nm. NO levels were measured in neonatal keratinocytes (**A**), endothelial cells (**B**), and fibroblasts (**C**). Exposed cell samples at each timepoint were accompanied by time-matched unexposed and positive controls. As experiments at individual timepoints were conducted independently, direct cross-comparison between timepoints was not performed. Across all cell types, visible light did not elicit significant increase in NO, indicating that low-level, full-spectrum sunlight-representative blue or red light does not modulate NO bioavailability. UV-B (1 J/cm^2^) served as positive control; statistical analysis was performed in GraphPad Prism™, version 10.1.2, using ordinary one-way ANOVA with mixed-effects model; significance was defined as *p* < 0.05. * = *p* < 0.05, ** = *p* < 0.01, *** = *p* < 0.001, **** = *p* < 0.0001.

When compared against unexposed controls for the corresponding timepoints in all experiments, no significant change in NO expression was visualised. This was noted in terms of whether skin cell lines were exposed to blue or red/infrared light (Figure 2A–C). Potential thermal effects that could influence the results were accounted for, minimising confounding due to heat exposure.

As expected, exposure to UV-B consistently induced a significant increase in fluorescence in both keratinocyte and endothelial cell lines when compared against the unexposed control for the timepoint in question. In contrast, fibroblasts exhibited a lower propensity for NO upregulation following UV-B exposure. These cells showed modest trends up to 30 min post-exposure, with variable (significant and non-significant) increases observed at later timepoints (Figure 2C). Importantly, foetal bovine serum (FBS) and phenol red were removed from the media prior to probe assessment to prevent interference.

## 4. Discussion

Our findings diverge from previous studies that report significant NO release following the exposure of cells to blue or red light. In non-skin-based cell types, Zhang et al. demonstrated this at 670 nm, observing NO induction following 7.5 J/cm^2^ exposure, while Rohringer et al. found NO induction at 24 J/cm^2^ of 516 nm or 635 nm light [25,26]. Considering this, it is feasible that skin cells (routinely exposed to visible light in vivo) may be more resistant to light-induced NO release than other cell types.

This interpretation is challenged by recent work from Barolet et al. and Albers et al., who demonstrate significant NO production in human skin cells following exposure to visible light [27,28]. Albers et al. suggest an effect on keratinocytes at 453 nm after 100 J/cm^2^ exposure [27], while Barolet uses a multi-wavelength LED array (455, 650, and 850 nm), delivering blue and red light at an irradiance of 20 mW/cm^2^ to achieve a dose of 15 J/cm^2^ over 12.5 min. Barolet’s study is particularly interesting given that the experimental setup closely matches our own in terms of the NO detection methodology (via use of DAF-FM diacetate) and incorporation of cell lines in part derived from donors of identical anatomical origin, age, and gender. Additionally, their study not only accounts for unwanted effects from thermal and cytotoxic insults (which may occur as part of light exposure regimens) through a ‘water-cooled irradiation system’ and viability assays but also goes one step further, validating the response from the probe through NO induction with the NO scavenger Carboxy-PTIO potassium salt (CPTIO). Here the researchers rule out other signalling intermediates such as ROS induced through oxidative stress, which is suggested to also interact with NO probes at a lower threshold [28].

It is likely that the delivery of visible light outside these specific narrow bands over much broader spectral ranges at levels representative for low-level terrestrial sunlight exposure in part explains the lack of response seen in the results presented here. Proof of this lies in Barolet’s study, where blue light exposures were applied at ~3 times higher doses when compared against our own work (4.86 J/cm^2^ vs. 15 J/cm^2^); this may be sufficient to initiate the photolysis of S-nitrosothiols (RSNOs) [28]. This is inferred as RSNO photolysis is tied to the blue light release of NO, owing to the fact that the metabolite requires lower energy than the photoreduction of nitrite (23–34 kcal/mol) [29,30,31]. Further supporting this interpretation is prior cited work in cutaneous and non-cutaneous cell types, including a study by Albers et al. who demonstrated NO release at 453 ± 10 nm, at doses upwards of 20 J/cm^2^ [29]. Together, these findings suggest that low-level exposures to sunlight may not be sufficient to elicit biologically meaningful NO production without UV (applied sun protection, diurnal or seasonal drop), raising important questions about the physiological plausibility of this mechanism under certain real-world conditions.

The reduced nitric oxide (NO) response observed in fibroblasts following UV-B exposure is an intriguing phenomenon that warrants further investigation in future UV-B-based studies. While the literature remains limited regarding nitrite availability and NO release in dermal cell lines when compared against epidermal cells [32], Opländer and Suschek report that nitrite depletion and excessive induction lead to greater toxicity and oxidative stress in fibroblasts in particular [33]. It is plausible then that the relatively narrow window required for fibroblast survival when compared against other cells present within the skins dermis and epidermis necessitates a lower threshold for NO release, thereby limiting NO bioavailability under UV-B exposure [33].

A consideration for future work is the addition of trace biological cofactors absent in the current model but present in whole tissue and blood. This is inferred given reports suggesting an interplay with protein- and non-protein-bound intermediates lost as part of the cell isolation process [29,30,34,35,36]. Opländer et al. highlight this for visible light exposures with the addition of copper (Cu) catalysing RSNO decomposition to NO under blue light exposure [30]. This is feasible in vivo, as both free copper and copper-containing enzymes may contribute to nitric oxide-related chemistry, although they represent distinct entities. Free copper ions are present within the epidermis at low micromolar concentrations (approximately 1–30 µM), whereas copper-containing enzymes such as ceruloplasmin circulate in plasma at slightly higher levels (12–25 µM) [30,37]. Similarly, Dejam et al. demonstrate that thiols lost during the purification of cell isolation and culture markedly enhance photolytic nitrate breakdown, increasing NO generation and RSNO formation [35]. Although these studies provide an interesting counterpoint to our own work and provide a mechanistic proof of principle, as experimental levels are supraphysiological (and likely enhance efficiency), it remains important to follow up with these claims under conditions approximating the environmental levels found in vivo. A case in point is Opländer’s work that utilises 100 µM copper chloride (CuCl_2_), greater than physiological levels [30,38].

The red and near-infrared light doses used in our study were broadly comparable to those reported by Barolet [28] but applied over broader spectral regions rather than specific narrow wavebands. Here, higher dose thresholds for wavelengths understood to interact with mitochondrial chromophores may be a factor [39,40]. These interactions may therefore lead to the photodissociation of NO that is transiently bound to cytochrome c oxidase, a key enzyme of the mitochondrial respiratory chain [40]. Thus, although we did not observe significant NO production under red light exposure, the broad-spectrum wavebands opted for over narrow wavebands may be a factor. Additionally, as mitochondrial NO may account for a small proportion of total NO production in some cells, such as endothelial cells, which possess only 2–5% of the mitochondrial content of other cell types relying primarily on glycolysis for ATP production [41], this may also be a factor explaining the results in this study.

A critical but often overlooked factor in in vitro studies of light-induced NO release is the medium used during irradiation. Several studies, including those by Albers, Rohringer, Zhang, and Oppländer, clearly state that cells were exposed in phosphate-buffered saline (PBS) [25,26,27,30,42]. However, many studies do not specify whether the culture medium was removed or replaced prior to light exposure, which is a significant omission, as media components such as tryptophan can degrade under light to generate ROS, RNS, or NO indirectly [43]. Without standardisation in exposure conditions, it becomes difficult to confidently interpret observed signals to direct NO production; thus even transient exposures from LEDs may have considerable effects on inflammatory pathways mediating ROS, RNS and NO induction.

Finally, although low-level visible light exposure from an artificial environmental source did not elicit a measurable increase in NO, other redox-dependent mediators, including low-level ROS such as hydrogen peroxide and carbon monoxide, may act in parallel or independently of NO [44,45,46,47]. These pathways have been shown in photobiomodulation studies to enhance antioxidant defences and reduce oxidative stress [43,44,45,46], suggesting that environmentally relevant visible light within low-level sunlight may subtly modulate redox homeostasis in skin and vascular cells in a comparable manner. Importantly, NO and other short-lived signalling mediators have a narrow therapeutic window and can promote oxidative stress at sub-optimum levels [47], highlighting the need for future in vivo studies to confirm whether light-induced redox changes within studies reporting positive data translate into beneficial local or systemic antioxidant effects.

## 5. Conclusions

The concept of visible-light-induced nitric oxide release remains of practical importance. While several studies have demonstrated NO generation using LED-based light sources, these findings have not been consistently replicated under conditions that reflect low-level environmental exposures. Such low-level exposures are relevant to mimic natural sunlight exposure while avoiding peak UV exposure. Discrepancies in exposure conditions including light source, dose, spectral power distribution and cell media may underlie the conflicting reported results.

Future work should aim to replicate these effects in more complex models that incorporate key biological cofactors, maintain native tissue architecture, and simulate environmental light conditions. This will be essential for an assessment of the potential for visible light to elicit meaningful NO-mediated effects outside of controlled clinical settings. In addition, further studies employing more sensitive methodologies may be warranted to determine whether lower-level NO upregulation occurs below DAF-FM and DAXJ2 probe-based detection thresholds. Alongside this, the incorporation of more sophisticated experimental models and detection methodologies may then follow the cross-talk between assessed cell lines and vascular smooth muscle cells, where downstream vasodilatory effects from NO are mediated via cGMP activation [48]. This may also provide further insight into the potential for visible light to induce blood pressure-lowering effects, which our work suggests needs to be treated with caution considering environmental doses.

## Data Availability

The datasets used and/or analysed during the current study are available from the corresponding author on reasonable request. All methods described above were performed in accordance with relevant guidelines and regulations.

## Supplementary Materials

The following supporting information can be downloaded at https://www.mdpi.com/article/10.3390/antiox15020232/s1. Table S1: Statistical analysis was conducted using GraphPad Prism™ using a two-way ANOVA, with Tukeys multiple comparisons test.

## Abbreviations

The following abbreviations are used in this manuscript:

NO	Nitric oxide
LED	Light-emitting diode
NOS	Nitric oxide synthase
UVR	Ultraviolet radiation
H_2_O_2_	Hydrogen peroxide
RNS	Reactive nitrogen species
CPTIO	Carboy PTIO potassium salt
ROS	Reactive oxygen species
RSNOs	S-nitrosothiols
Cu	Copper
CuCl_2_	Copper chloride
FBS	Foetal bovine serum
PBM	Photobiomodulation
